# Brain microvascular endothelial-astrocyte cell responses following Japanese encephalitis virus infection in an *in vitro* human blood-brain barrier model

**DOI:** 10.1016/j.mcn.2018.04.002

**Published:** 2018-06

**Authors:** Adjanie Patabendige, Benedict D. Michael, Alister G. Craig, Tom Solomon

**Affiliations:** a The Institute of Infection and Global Health, University of Liverpool, Liverpool, UK; b The School of Biomedical Sciences and Pharmacy, The University of Newcastle, Newcastle, Australia; c The Hunter Medical Research Institute, Newcastle, Australia; d The Walton Centre NHS Foundation Trust, Liverpool, UK; e Center for Immunology and Inflammatory Disease, Massachusetts General Hospital, Harvard Medical School, USA; f Liverpool School of Tropical Medicine, Liverpool, UK; g National Institute for Health Research Health Protection Research Unit in Emerging and Zoonotic Infections, Liverpool, UK

**Keywords:** BBB, blood-brain barrier, CCL, C-C motif chemokine ligand, CXCL, C-X-C motif chemokine ligand, Dex, dexamethasone, DPI, days post infection, G-CSF, granulocyte-colony stimulating factor, GM-CSF, granulocyte macrophage-colony stimulating factor, HBECs, human brain endothelial cells, ICAM1, intercellular adhesion molecule-1, IFN, interferon, IL, interleukin, JE, Japanese encephalitis, JEV, Japanese encephalitis virus, MMP, matrix metalloproteinase, MOI, multiplicity of infection, MPO, myeloperoxidase, TEER, transendothelial electrical resistance, TNF, tumour necrosis factor, TRAIL, TNF-apoptosis-inducing ligand, VCAM, vascular cell adhesion molecule, VEGF, vascular endothelial growth factor, WNV, West Nile virus, Blood-brain barrier, *In vitro* models, Japanese encephalitis virus, Viral encephalitis, Dexamethasone, TEER

## Abstract

Japanese encephalitis virus (JEV) remains a leading cause of encephalitis, globally, which continues to grow in importance despite the availability of vaccines. Viral entry into the brain can occur *via* the blood-brain barrier (BBB), and inflammation at the BBB is a common final pathway in many brain infections. However, the role of the BBB during JEV infection and the contribution of the endothelial and astrocytic cell inflammation in facilitating virus entry into the brain are incompletely understood. We established a BBB model using human brain endothelial cells (HBECs) and human astrocytes. HBECs are polarised, and therefore the model was inoculated by JEV from the apical side to simulate the *in vivo* situation. The effects of JEV on the BBB permeability and release of inflammatory mediators from both apical and basolateral sides, representing the blood and the brain side respectively were investigated. JEV infected HBECs with limited active virus production, before crossing the BBB and infecting astrocytes. Control of JEV production by HBECs was associated with a significant increase in permeability, and with elevation of many host mediators, including cytokines, chemokines, cellular adhesion molecules, and matrix metalloproteases. When compared to the controls, significantly higher amounts of mediators were released from the apical side as opposed to the basolateral side. The increased release of mediators over time also correlated with increased BBB permeability. Treatment with dexamethasone led to a significant reduction in the release of interleukin 6 (IL6), C-C motif chemokine ligand 5 (CCL5) and C-X-C motif chemokine ligand 10 (CXCL10) from the apical side with a reduction in BBB disruption and no change in JEV production. The results are consistent with the hypothesis that JEV infection of the BBB triggers the production of a range of host mediators from both endothelial cells and astrocytes, which control JEV production but disrupt BBB integrity thus allowing virus entry into the brain. Dexamethasone treatment controlled the host response and limited BBB disruption in the model without increasing JEV production, supporting a re-investigation of its use therapeutically.

## Highlights

• Japanese encephalitis virus (JEV) infects human brain endothelial cells (HBECs).
• This triggers the production of a range of host mediators from both HBECs and astrocytes.
• JEV infection adversely affects blood-brain barrier (BBB) integrity.
• Dexamethasone treatment following JEV infection reduces the inflammation.
• Dexamethasone restores BBB integrity without increasing the levels of JEV particles.

## 1 Introduction

Encephalitis is inflammation and swelling of the brain, which can be caused by a range of viruses, often with devastating results (Solomon et al., 2014). Globally JEV is one of the most important causes. Although largely confined to South and East Asia, this mosquito-borne flavivirus (genus *Flavivirus*, family *Flaviviridae*) has spread to the northern territories of Australia and potentially to European countries with the recent discovery of JEV RNA in mosquitoes in northern Italy (Ravanini et al., 2012). The virus is closely related to West Nile virus (WNV) and Zika virus; the former causes encephalitis in a small proportion of patients; the latter has caused large outbreaks of febrile illness and congenital brain disease in Latin America (Vasilakis & Weaver, 2017). There are an estimated 67,000 cases of Japanese encephalitis (JE) annually with 13–24,000 deaths (Campbell et al., 2011). In addition, more than half the survivors have severe neurological sequelae, posing a large economic and health burden on the communities affected (Gould & Solomon, 2008). Vaccines are available for JEV, but their use is limited by cost and availability, especially in many low-middle income countries where JEV is endemic (McArthur & Holbrook, 2011). There is currently no specific treatment for JEV or indeed any flavivirus. Dexamethasone is sometimes used to control brain swelling, although a small trial in Thailand failed to show any benefit (Hoke et al., 1992). Furthermore, corticosteroids such as hydrocortisone and dexamethasone in low doses (nM range) have also been shown to improve BBB integrity *in vivo* (Salvador et al., 2014) and *in vitro* (Deli et al., 2005).

JEV is transmitted naturally in an enzootic cycle between birds, pigs, and other vertebrate hosts by *Culex* mosquitoes. Humans are dead-end hosts. Following inoculation during the bite of a blood-feeding mosquito, JEV is thought to replicate in the skin and local lymph nodes, causing a transient low viraemia, before crossing the BBB to gain entry into the central nervous system (CNS) (Turtle & Solomon, 2012). How exactly JEV crosses the BBB to enter the CNS is not known, but three routes are possible: passive transport of whole virions across the endothelium, replication of virus within the endothelium, or a “Trojan horse” mechanism (diapedesis) of infected leukocytes between endothelial cells (Solomon & Vaughn, 2002). BBB damage due to inflammation is thought to be a common final pathway in many brain infections, allowing pathogens and immune cells access into the brain, and leading to CNS damage (Miner & Diamond, 2016). At autopsy, histopathological changes of inflammation, including BBB breakdown and infiltration of leukocytes are common in JE, and the anti-inflammatory corticosteroid drug dexamethasone is sometimes given in an attempt to control this (Hoke et al., 1992). However, the extent to which this inflammatory response actually contributes to viral entry across the human BBB is unclear. A better understanding of the means by which JEV crosses the BBB, and the contribution of the host response to this may point the way towards developing new therapeutic approaches not only for JEV but also for similar flaviviruses such as WNV and Zika virus.

Although animal models can provide useful insights into the pathogenesis of encephalitis (Liu et al., 2008; German et al., 2006; Mishra et al., 2009; Myint et al., 1999), their applicability to the human BBB is limited. There are considerable species-specific differences in BBB, including different expression levels and functionalities of BBB transporters, differences in BBB metabolic enzyme activities, amyloid-beta clearance rates, and timing of BBB maturation, to name a few (Vogelgesang & Jedlitschky, 2014; Qosa et al., 2014; Syvänen et al., 2009; Chu et al., 2013; Deo et al., 2013; Semple et al., 2013). *In vitro* models of the human BBB provide greater flexibility for studying the BBB in detail, allow mechanistic insight into the cross talk between virus and the brain endothelium, and give additional physiologically relevant human data, while reducing the reliance on animal models. Cultured human brain endothelial cells tend to lose some BBB features (*e.g.* reduced expression of tight junction proteins, transporters, receptors and enzymes) after extraction, but most can be induced by using specialised growth medium containing soluble differentiation factors and by co-culturing with astrocytes and/or pericytes (Patabendige, 2012). We therefore established a co-culture model of the human BBB (Ferguson et al., 2015) using a well-characterised human brain endothelial cell line (Wassmer et al., 2006) and primary human astrocytes. Using this model, we examined the effects of JEV infection in HBECs and the associated pro-inflammatory host response on the BBB integrity, and investigated the impact of the corticosteroid drug dexamethasone on these parameters.

## 2 Material and methods

### 2.1 Establishment of the *in vitro* human BBB model

HBECs were kindly provided by Georges Grau (University of Sydney Medical School) and were used up to passage 20 (Wassmer et al., 2006). HBECs were grown in collagen-coated flasks and maintained in Dulbecco's Modified Eagle's Medium (DMEM) containing 2 mM glutamine, 100 U/ml penicillin, 100 μg/ml streptomycin, and 550 nM hydrocortisone (Sigma, UK), 10% fetal bovine serum (FBS) and human recombinant epidermal growth factor (First Link, UK). Primary human astrocytes were purchased from ScienCell (USA) and were used at passage 2–5. Human astrocytes were grown in flaks coated with poly-d-lysine and were maintained in DMEM/Ham's Nutrient Mixture F12 (Sigma, UK) containing 10% FBS, 2 mM glutamine and 100 U/ml penicillin, 100 μg/ml streptomycin. Cells were maintained in 5% CO_2_ humidified atmosphere at 37°C and were passaged every 3–4 days and the culture medium replaced every 2–3 days. To set up the co-culture BBB model, HBECs were seeded on collagen-coated Transwell-Clear inserts (Corning, USA) at 1.0 × 10^5^ cells/insert (0.4 μm pores, 1 mm diameter, 0.5 ml) and were transferred to a 12-well plate containing confluent human astrocytes (1.0 × 10^5^ cells/well, 1.5 ml) as described previously (Ferguson et al., 2015). Experiments were performed when HBECs were confluent and when transendothelial electrical resistance (TEER, a functional measurement of BBB integrity) of the model was over 50 Ω·cm^2^.

### 2.2 Transendothelial electrical resistance measurements

TEER across the BBB model was determined using STX100C electrodes connected to an EVOM2 epithelial voltohmmeter (World Precision Instruments, UK) as described previously (Patabendige & Abbott, 2014; Patabendige et al., 2013a). The resistance of cells grown on Transwell filter inserts was corrected for resistance across a cell-free insert, and multiplied by surface area, to give TEER in ohms x cm^2^ (Ω·cm^2^).

### 2.3 Infecting cells with JEV

A strain of JEV (CNS138-11, GenBank accession no: AY184213) isolated from the brain of a fatal human case in 1999 from Sarawak, Malaysian Borneo was used for all experiments (Solomon et al., 2003). JEV was propagated in Vero cells, harvested at 5–6 days post inoculation, and titrated by plaque formation in Vero cells. In brief, six well plates were inoculated with 10-fold dilutions of JEV and left to adsorb for 1 h before an agarose overlay of Minimum Essential medium (MP Biomedicals, UK) containing 5% FBS was added. The cells were incubated at 37 °C for 5–6 days, fixed, and plaques were visualised by crystal violet staining. The BBB model was infected with JEV from the apical (blood) side at a multiplicity of infection (MOI) of 1 for one hour and fed with fresh media. Virus diluent media (DMEM with 2% FBS) was used for mock-infected controls. Samples were taken immediately post infection and at serial time points from both the apical and basolateral (brain) sides for cytokine analysis or plaque assays. TEER was measured before infection and then at serial time points post infection. To determine the effectiveness of the human BBB model in restricting JEV entry, the Transwell system was set up as described in co-cultures, monocultures of either HBECs only or human astrocytes only and cell-free inserts. The system was inoculated with JEV (MOI = 1) from the apical side and samples were taken at serial time points post infection from both apical and basolateral sides. For the JEV growth curve, HBECs were infected as described above and supernatants were collected at 0–4 days post infection. Infectious virus particles were quantified by plaque assay on Vero cells.

### 2.4 Transmission electron microscopy

For transmission electron microscopy, JEV-infected cells were fixed with 5% glutaraldehyde in 0.1 M sodium cacodylate. Pellets were then fixed in 1% Osmium tetroxide, stained with Uranyl acetate, dehydrated in a series of ethanols, acetone, infiltrated with araldite resin and polymerised at 60 °C. Ultrathin Sections (60–90 nm) were cut with ‘Diatome’ diamond knife on ‘Reichert-Jung Ultracut’ ultramicrotome (Leica Microsystems, UK) and mounted on 200 mesh copper grids and stained with “Reynold's Lead citrate” stain. Images were captured using Phillips EM208S transmission electron microscope at 80KV. Standard EM/laboratory reagents and material were from TAAB Laboratories Equipment Ltd., UK and Sigma.

### 2.5 Cytometric magnetic-bead array

Supernatants were taken from JEV-infected and mock-infected controls from the apical and basolateral sides at 0, 6, 24 and 48 h post infection. The inflammatory mediators were assessed using a commercial cytometric magnetic-bead array assay system (Procarta Immunoassay kit, Panomic Solutions, Affymetrix Milano, Italy) in accordance with the manufacturer's instructions. The mediators assessed were identified from previous literature; granulocyte-colony stimulating factor (G-CSF), granulocyte macrophage-colony stimulating factor (GM-CSF), interleukin (IL) 1α, IL1β, IL1-RA, IL4, IL6, IL10, IL13, IL17a, IL17f, CXCL 8 (IL8), CXCL9, CXCL10, CCL2, CCL3, CCL4, CCL5, CCL11, myeloperoxidase (MPO) Sap/Pentraxin 2, and tumour necrosis factor (TNF), TNF-apoptosis-inducing ligand (TRAIL), interferon (IFN) α2, β, γ, ο, leptin, vascular endothelial growth factor (VEGF), eSelectin, granzyme B, intercellular adhesion molecule-1 (ICAM1), vascular cell adhesion molecule (VCAM), matrix metalloproteinase (MMP)7, MMP8 and MMP9. Fluorescence intensity was determined using a Bio-Rad platform (BioPlex Manager 4.1, Bio-Rad Laboratories, UK). Standards and samples (two independent experiments in triplicates) were analysed in duplicate and the mean value used in analysis. Control samples were pooled and analysed in duplicates. Standard curves were adjusted at the points of fluorescence intensity saturation, generating a sigmoid curve with 6–8 points of fluorescence intensity, as described previously (Michael et al., 2013). To avoid undetectable levels of mediators biasing the analysis, only mediators detected in at least 80% of the samples were included in the analysis, as described previously (Michael et al., 2016). The results were normalised to volume of the compartments to give units in picogram (pg).

### 2.6 Dexamethasone treatment

Dexamethasone was purchased from Sigma, UK. TEER was measured in the BBB model as described above and infected with JEV (MOI = 1) or mock-infected from the apical side. Cells were then treated with 2 μg/ml dexamethasone. TEER was measured and samples from both apical and basolateral sides were taken for analysis (*n* = 3) by a custom Multi-Analyte ELISArray kit from SABiosciences (Qiagen, UK) for IL6, CCL5 and CXCL10 levels according to the manufacturer's instructions. This kit allows a rapid assessment of the level of multiple cytokines and chemokines in multiple samples over one standardised development time. Absorbance reading at 450 nm was subtracted from the absorbance reading at 570 nm to correct for artificially high signals. The linear range of the assay is optical density readings between 0 and 2.5 at 450 nm for all analytes.

Samples from both apical and basolateral were also taken for analysis by plaque assay to determine JEV production as described earlier.

### 2.7 Statistical analysis

Data were expressed as mean ± standard error of the mean (SEM) or mean ± standard deviation (SD) and analysed and presented using Microsoft Excel 2007, 2010, or GraphPad Prism (versions 6 and 7) software. Groups of two were analysed using Student's *t*-test, and two-way ANOVA was used to analyse two independent variables. Values were considered to be significantly different when the probability <5% (*p* < 0.05). All experiments were repeated at least 2–4 times. Overall cytometric bead array mediator data from control and JEV infected groups underwent a one-way hierarchical cluster analysis. Then mediator data from each group underwent Pearson's correlation nearest neighbour analysis generating proximity matrices, using SPSS (SPSS Inc., 2011); from these matrices, heat-maps were generated using SigmaPlot (SigmaPlot, Systat Software Inc., USA); extraction heat-maps were then generated by overlaying the proximity matrix from the JEV infected model over the control, as described previously (Michael et al., 2013). This generates a visual representation of the interaction of all mediators and the extraction heat-map represents the differences between the two groups.

## 3 Results

### 3.1 JEV infects both human brain endothelial cells and astrocytes, and increases BBB permeability with limited active viral production in endothelial cells

**Fig. 1:**
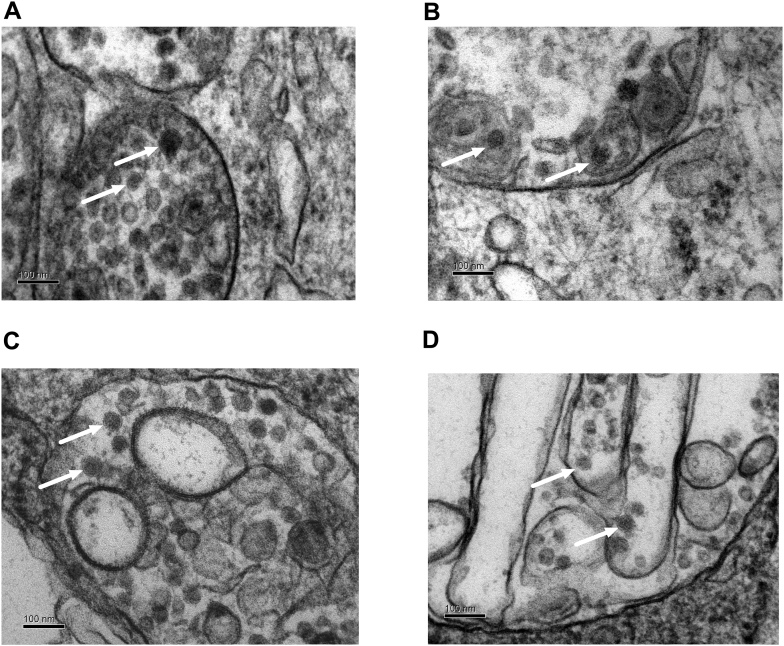
JEV is able to infect the cells forming the BBB. Confluent monocultures of HBECs and human astrocytes were infected with JEV and fixed at 3 days post infection (dpi) at MOI = 1. Using electron microscopy, JEV particles (arrows) were detected in cytoplasmic vacuoles in both HBECs (A–B) and human astrocytes (C–D); most were hexagonal in shape and 40–50 nm diameter with an electron dense core and an outer membrane (scale bar = 100 nm).

Using electron microscopy, we were able to detect JEV particles (40–50 nm diameter) in JEV-infected monocultures of HBECs (Fig. 1A) and in human astrocytes (Fig. 1B) three days post infection (dpi). Immunofluorescence experiments using an antibody against the JEV E glycoprotein also confirmed that JEV is able to infect HBECs and astrocytes (Supplementary Fig. 1).

**Fig. 2:**
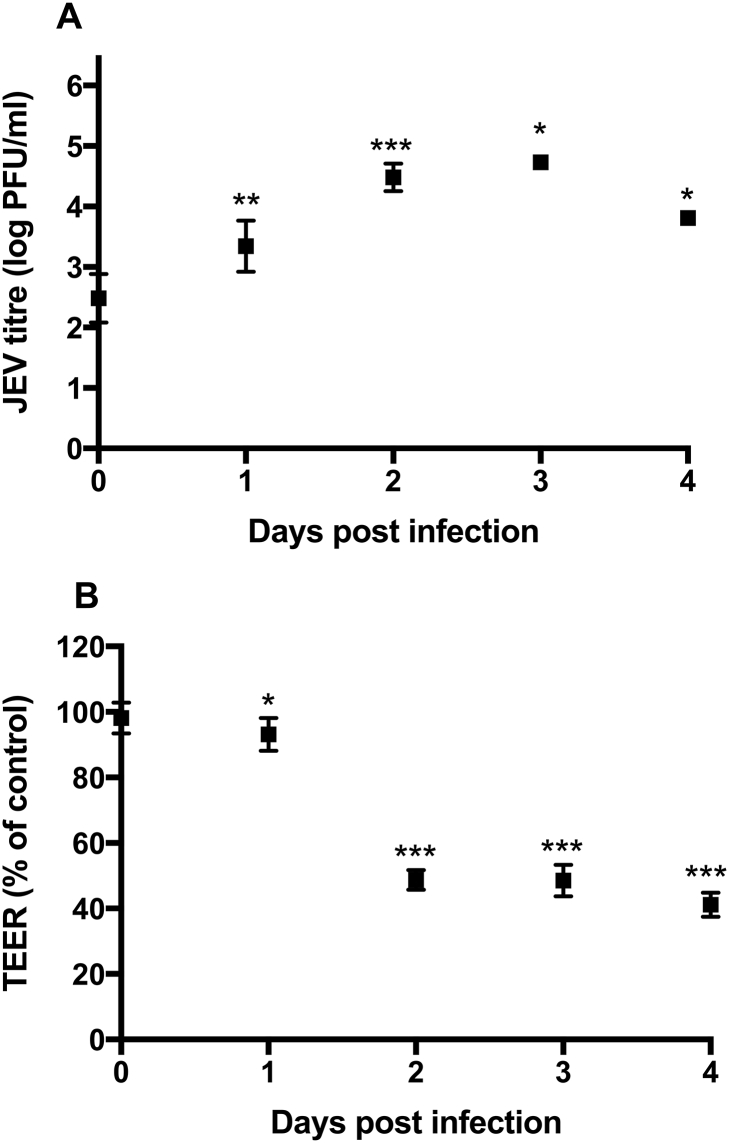
JEV is able to infect human brain endothelial cells with limited active virus production, and increases BBB permeability. (A) JEV growth curve in HBECs: HBEC monocultures were infected with JEV (MOI = 1) and samples were collected at serial time points from the apical side for analysis by plaque assay. (B) BBB integrity was assessed by measuring transendothelial electrical resistance (TEER) in the BBB model before infection (dpi = 0) and then at serial time points following JEV infection (MOI = 1). Only cultures with TEER over 50 Ω·cm^2^ were used. Results are mean TEER (% of control at each time point ± SD). All experiments are *n* = 2–3; student's *t*-Test, compared to day 0; *P < 0.05, *P < 0.01, ***P < 0.001.

Firstly, we assessed the virus production in monocultures of HBECs. Plaque assays showed limited active viral production in HBECs (highest JEV titre up to 1 × 10^5^ PFU/ml in HBECs *cf.* 1 × 10^7^ in Vero cells, where JEV was propagated). After an initial significant rise in the plaque forming units (PFU) at 2 dpi, the PFU declined following 3 dpi (Fig. 2A). Therefore, we assessed the BBB permeability up to 4 dpi in the co-culture model. Infection of the co-culture human BBB model with JEV from the apical side led to a significant drop in TEER 2 dpi (*p* < 0.001), and remained at this level (Fig. 2B), indicating that BBB integrity is lost early during infection, and the BBB permeability is not increased despite further viral production within the endothelium.

### 3.2 Human brain endothelial cells act as an initial barrier to JEV transmigration

**Fig. 3:**
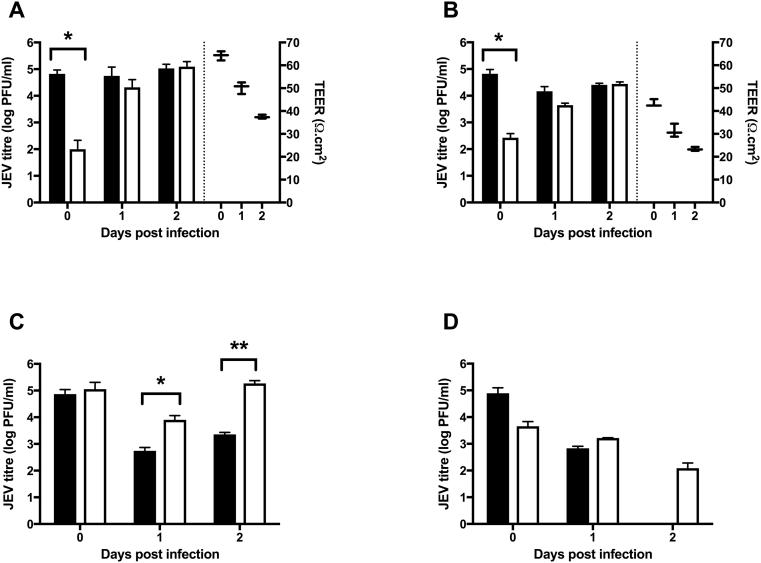
Barrier properties of the human BBB model following JEV infection. To assess the effectiveness of the barrier properties of the BBB model and to quantify JEV production and cross over, Transwells were set up as (A) co-cultures with HBECs grown on the inserts with astrocytes in the well, (B) monocultures of either HBECs only on the inserts or (C) astrocytes only in the well with a cell-free insert on top, and (D) cell-free inserts/wells as blanks. The apical side was inoculated with JEV (MOI = 1). TEER (box and whiskers) was measured first, and then samples were taken at serial time points from both sides for analysis by plaque assay. Results are mean ± SD (*n* = 3). Student's *t-*Test, comparing apical (black bars) and basolateral (white bars) sides; *P < 0.05, **P < 0.01.

We then assessed the barrier properties of HBECs to JEV transmigration up to 2 dpi, given JEV titre/production significantly increase by 2 dpi, and TEER does not decrease further after 2 dpi. Significantly low levels of infectious JEV particles were detected in the basolateral sides of the culture systems with HBECs (Fig. 3A–B) compared to cultures with astrocytes only (Fig. 3C) or in the cell-free inserts (Fig. 3D) immediately post the inoculation period, indicating a barrier to JEV entry to the basolateral compartment by the HBEC monolayer (Fig. 3). This was further confirmed by real-time RT-PCR experiments, where significantly lower amounts of viral mRNA (*P* < 0.01) were found on the basolateral side compared to the apical side (Supplementary Fig. 2). However, there was no significant difference between apical and basolateral levels of JEV in cultures with HBECs after 1–2 days post infection (Fig. 3). Significantly higher levels of JEV were detected 1–2 days post infection in astrocyte only cultures (Fig. 3C) on the basolateral side compared to the apical side, indicating that JEV is able to infect and replicate in human astrocytes. Not surprisingly, JEV production continued to decline in the cell-free inserts after initial inoculation (Fig. 3D). Corresponding TEER data further demonstrate the barrier properties of HBECs. Co-culture with human astrocytes strengthened the barrier properties of HBECs, though TEER continued to decline in both systems gradually (Fig. 3 A–B).

### 3.3 JEV infection of the BBB model induces a strong pro-inflammatory response from HBECs and astrocytes, with a significant increase in mediator release from the apical side compared to the basolateral side

**Fig. 4:**
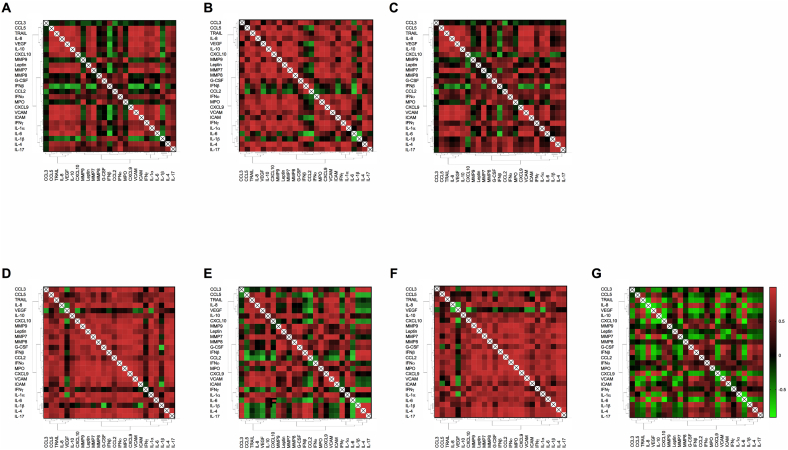
JEV infection of the BBB induces a strong pro-inflammatory response from host cells. The BBB model was infected with JEV (MOI = 1) from the apical side and supernatants were collected at serial time points from both apical and basolateral sides for analysis by cytometric bead array to detect inflammatory mediators (n = 2). Heat-maps of nearest-neighbour correlations of cytokines and chemokines for the (A) JEV basolateral, (B) control basolateral, (C) basolateral JEV-control extraction, (D) JEV apical, (E) control apical, (F) apical JEV-control extraction, and (G) JEV apical-basolateral extraction heat-maps. Red indicates a positive correlation (+1), green a negative correlation (−1), and black as no correlation (0). Therefore, whilst the diagonal white line simply represents the correlation of each mediator with itself (*i.e.* +1) that is excluded from analysis, the rest of the heatmap provides a visual representation of the positive or negative correlations of each mediator with each other.

Statistically significant elevations in the levels of many inflammatory mediators were found in both the apical and basolateral side of the JEV infected BBB model in comparison to the control (Table 1). In addition, when compared to the controls, many mediators were detected at significantly higher levels on the apical as opposed to basolateral side. The majority of mediators demonstrated strong positive correlations with each other in both the JEV infected model and the control, indicated in red on the heat-maps, suggesting a broad up-regulation of the production of all host-inflammatory mediators assessed in concert (Fig. 4.). However, the extraction heat-maps demonstrated that, in samples from the apical side as opposed to the basolateral side, many more mediators had positive correlations in the JEV infected model than the control, suggesting greater co-ordinated production of these host-inflammatory mediators in the apical compartment. Nevertheless, even on the basolateral side there were two broad clusters that correlated positively within and between clusters, the first included CCL5, VEGF, and CXCL10, and the second cluster included VCAM, ICAM, and IL-6. Interestingly, in the extraction heat-map of JEV-infected apical and JEV-infected basolateral samples, there were several smaller clusters. One included CXCX10 and MMP9 which had several positive correlations, including with IL-1α. Another included G-CSF and CCL2, and this had positive correlations with IL-1β. IL-6 had many negative correlations, but had positive correlations with MMP7 and VEGF, suggesting that in those samples IL-6 might be being up-regulated in co-ordination with these other mediators.

**Table 1:** Comparison of inflammatory mediators released in control and JEV-infected human BBB models over 48 h.

Mediator	Basolateral (pg)			Apical (pg)			Basolateral *vs.* Apical JEV
	JEV mean (SD)	Control mean (SD)	JEV *vs.* control *P* value	JEV mean (SD)	Control mean (SD)	JEV *vs.* control P value	P value
GCSF	24.47 (2.43)	10.62 (1.44)	<0.001	13.64 (6.68)	3.988 (0.70)	<0.05	<0.001
IFNβ	32.93 (5.84)	16.26 (1.79)	<0.001	12.74 (1.05)	6.352 (1.85)	<0.001	<0.001
IFNο	1.325 (1.78)	0.6366 (0.22)	<0.001	0.4259 (0.04)	0.2371 (0.09)	<0.001	<0.001
IL1α	8.498 (5.37)	2.924 (1.81)	NS	9.634 (7.77)	2.185 (0.75)	NS	NS
IL1β	9.292 (1.65)	4.607 (1.03)	<0.001	3.581 (0.38)	1.766 (0.50)	<0.001	<0.001
IL4	12.09 (0.83)	5.874 (0.99)	NS	5.093 (0.99)	2.442 (0.66)	<0.001	<0.001
IL6	5018 (3577)	2110 (787.6)	NS	3497 (1026)	360.2 (217.7)	<0.001	NS
IL8	5944 (6388)	1984 (638.5)	NS	9242 (9393)	484.3 (182)	NS	NS
IL10	2.035 (0.46)	0.9175 (0.18)	<0.001	1.037 (0.16)	0.2782 (0.04)	<0.001	<0.001
CXCL 10	126.6 (163.8)	33.75 (37.2)	NS	209.8 (220.5)	34.27 (31.17)	NS	NS
CCL2	10,113 (4855)	5860 (4194)	NS	4503 (2221)	1202 (240)	<0.05	<0.05
CXCL9	5.178 (2.45)	2.436 (0.57)	NS	2.452 (0.92)	0.6154 (0.13)	<0.05	<0.05
CCL3	6.362 (1.41)	2.697 (0.34)	<0.001	3.096 (1.05)	1.292 (0.44)	<0.05	<0.001
CCL5	643.5 (371.8)	366.4 (167.2)	NS	744.9 (278.1)	264.8 (167.9)	<0.05	NS
TRAIL	267.1 (115.9)	125 (26.72)	<0.05	162.3 (41.73)	34.19 (9.25)	<0.001	<0.05
VEGF	1354 (887.8)	639.1 (353)	NS	1301 (282.5)	148.7 (94.37)	<0.001	NS
ICAM	516.6 (305.8)	284.3 (126.9)	NS	621.3 (487.3)	249 (160)	NS	NS
IFNο	1.186 (0.48)	0.7116 (0.29)	NS	0.4948 (0.18)	0.1637 (0.03)	<0.05	<0.05
MPO	78.09 (66.68)	25.37 (4.61)	NS	22.18 (4.68)	8.993 (2.09)	<0.001	<0.05
VCAM	89.95 (30.26)	65.34 (15.88)	NS	74.52 (51.29)	34.97 (14.33)	NS	NS
MMP7	235 (109.1)	124.6 (47.26)	NS	85.52 (26.78)	30.66 (10.17)	<0.05	<0.05
MMP8	105.1 (87.56)	39.33 (8.33)	NS	32.94 (8.41)	12 (3.97)	<0.001	<0.05
MMP9	6.226 (6.96)	2.047 (0.40)	NS	1.762 (0.37)	0.7931 (0.17)	<0.001	NS
Leptin	98.38 (27.72)	41.79 (43.18)	<0.05	31.93 17.66)	12.62 (6.22)	NS	<0.001
IL17	6.457 (0.57)	3.249 (0.30)	<0.001	2.529 (0.57)	1.45 (0.71)	<0.05	<0.001

### 3.4 Release of inflammatory mediators following JEV infection increases with time and correlates with TEER

**Fig. 5:**
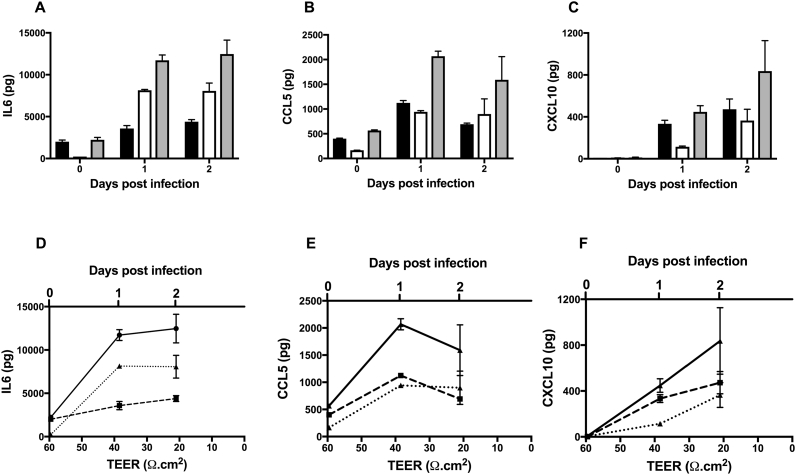
JEV Infection increases the release of several inflammatory mediators over time and correlates with TEER. The BBB model was infected with JEV (MOI = 1) from the apical side, TEER measured and supernatants were collected at serial time points (0–2 dpi) from both apical (black bars) and basolateral (white bars) sides for analysis by cytometric bead array. Grey bars indicate total amounts. JEV Infection significantly increased the release of many mediators including, (A) IL6, (B) CCL5 and (C) CXCL10 over time (*n* = 2; two-way ANOVA *P* < 0.001 for all three mediators). Correlation of the release of (D) IL6, (E) CCL5 and (F) CXCL10 from apical (dashed line), and basolateral (dotted line) sides, and the total amounts (continuous line) with TEER at the corresponding serial time points as shown for A-C (n = 2).

Several mediators, including IL6 (Fig. 5A), CCL5 (Fig. 5B) and CXCL10 (Fig. 5C) significantly increased with time following JEV infection (two-way ANOVA; *P* < 0.001 for all three mediators). Many of the mediators identified from the apical side were found to correlate with TEER, including IL-6, CXCL10, VCAM and MMP7 (Table 2, Fig. 5D and F). Fewer mediators were identified on the basolateral side to correlate with TEER. Some mediators may cross the barrier as it becomes increasingly permeable and therefore correlation with TEER may reflect this. Interestingly, excluding mediators present at higher levels on the apical side, the mediators identified on the basolateral side that had a significant correlation with TEER were IL-6, CXCL10, VEGF, ICAM and VCAM. In addition, to account for the potential for mediators to cross the barrier as it becomes more permeable, we also measured the total mediator level from both sides of the barrier and analysed for any correlations with TEER. Significant correlations with TEER were found for many mediators including IL-6, CXCL10, VCAM, MMP7 and leptin.

**Table 2:** Significant correlations of inflammatory mediators with TEER following JEV infection.

Mediator	Basolateral	*P* value	Apical	*P* value	Total	*P* value
	Pearson's correlation		Pearson's correlation		Pearson's correlation	
GCSF	−0.015	NS	−0.925	<0.01	−0.886	<0.01
IFNβ	−0.769	<0.05	−0.781	<0.05	−0.708	<0.05
IFNγ	−0.235	NS	−0.167	NS	−0.244	NS
IL1α	−0.501	NS	−0.865	<0.01	−0.821	<0.05
IL1β	−0.579	NS	−0.004	NS	−0.432	NS
IL4	−0.327	NS	−0.815	<0.05	−0.638	NS
IL6	−0.775	<0.05^a^	−0.917	<0.01	−0.826	<0.05
IL8	−0.525	NS	−0.838	<0.05	−0.732	<0.05
IL10	−0.732	<0.05	−0.837	<0.05	−0.824	<0.05
CXCL 10	−0.789	<0.05^a^	−0.889	<0.01	−0.887	<0.01
CCL2	0.000	NS	−0.294	NS	−0.083	NS
CXCL9	−0.819	<0.05	−0.918	<0.01	−0.864	<0.01
CCL3	−0.239	NS	−0.793	<0.05	−0.084	NS
CCL5	−0.605	NS	−0.201	NS	−0.458	NS
TRAIL	−0.713	<0.05	−0.555	NS	−0.695	<0.05
VEGF	−0.868	<0.01^a^	−0.509	NS	−0.906	<0.01
ICAM	−0.683	<0.05^a^	−0.910	<0.01	−0.933	<0.01
IFNο	−0.092	NS	−0.772	<0.05	−0.250	NS
MPO	−0.014	NS	−0.865	<0.01	−0.167	NS
VCAM	−0.723	<0.05^a^	−0.973	<0.001	−0.913	<0.01
MMP7	−0.823	<0.05	−0.966	<0.001	−0.879	<0.01
MMP8	−0.386	NS	−0.781	<0.05	−0.562	NS
MMP9	−0.409	NS	−0.733	<0.05	−0.041	NS
Leptin	−0.878	<0.01	−0.888	<0.01	0.955	<0.001
IL17a	−0.037	NS	−0.914	<0.01	−0.479	NS

a Mediators not present at greater concentrations in samples from the apical side that demonstrated significant basolateral correlations with TEER.

### 3.5 Dexamethasone treatment dampens the immune response and preserves barrier integrity without significant changes in infectious JEV levels

**Fig. 6:**
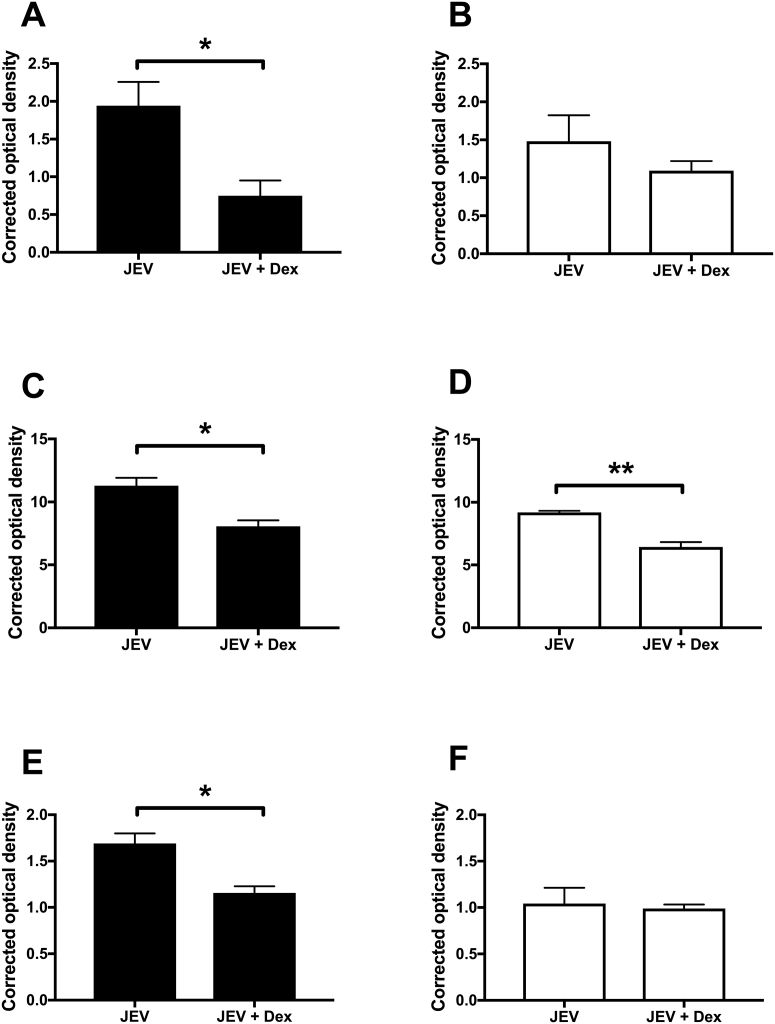
Dexamethasone treatment dampens the immune response. The co-culture human BBB model was infected from the apical side with JEV (MOI = 1). Some cultures were treated with 2 μg/ml dexamethasone (Dex) from the apical side immediately post infection. Supernatants were collected from both apical (black bars) and basolateral (white bars) side at 2 dpi for analysis by Multi-Analyte ELISArray for IL6 (A-B), CCL5 (C–D) and CXCL10 (E–F) levels. Results are mean ± SE (*n* = 3), Student's *t-*Test, comparing JEV and JEV + Dex; *P < 0.05, **P < 0.01.

**Fig. 7:**
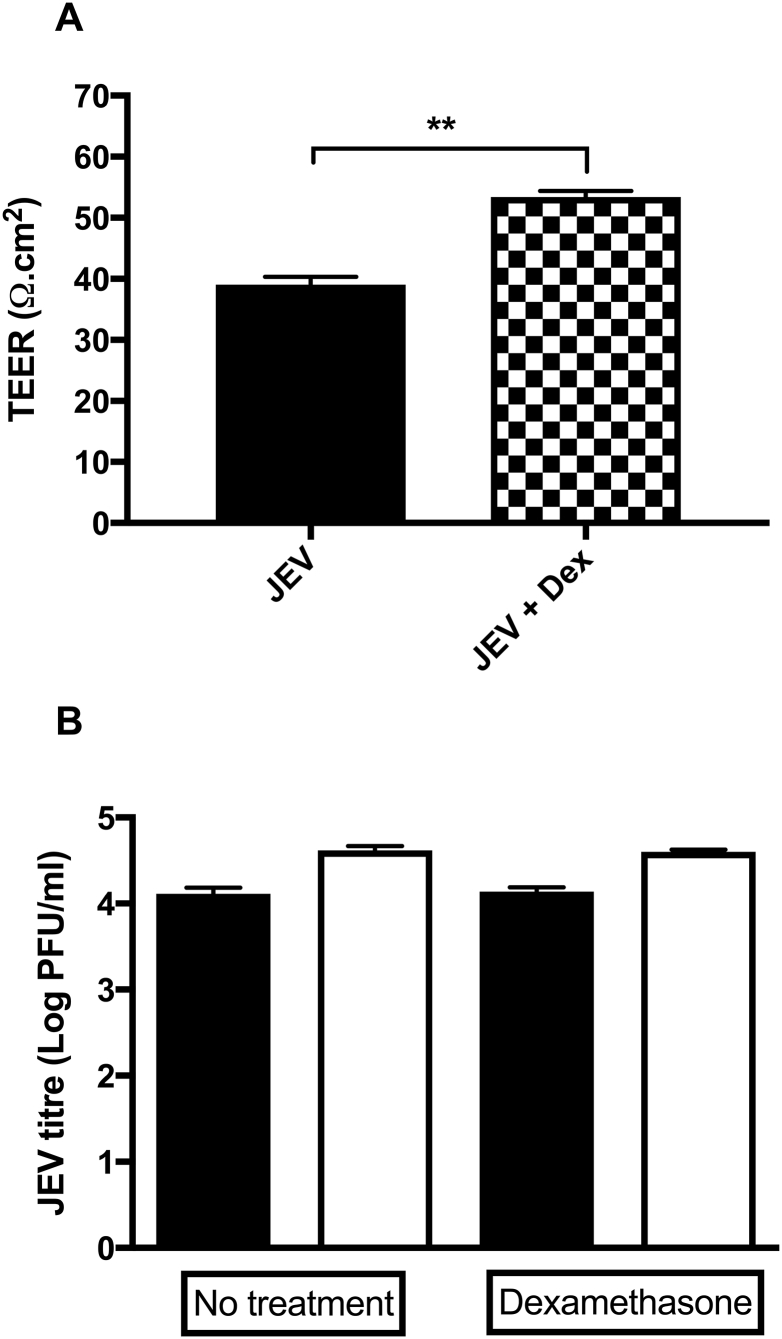
Dexamethasone treatment preserves barrier integrity and does not affect JEV production. The co-culture human BBB model was infected from the apical side with JEV (MOI = 1). Some cultures were treated with 2 μg/ml dexamethasone (Dex) from the apical side immediately post infection. (A) TEER was measured to assess the effects of Dexamethasone on BBB integrity. Results are mean ± SE (n = 3), Student's *t-*Test, comparing JEV and JEV + Dex; **P < 0.01. (B) Supernatants were collected from both apical (black bars) and basolateral (white bars) sides at 2 dpi for analysis by plaque assay to assess JEV production. Student's *t-*Test, comparing No treatment and Dex treatment; *P* = 0.7 for both apical and basolateral JEV titres.

JEV infection led to a significant increase in the levels of IL6, CCL5, and CXCL10 in both apical and basolateral sides of the JEV infected BBB model compared to the control (*p* < 0.05 to *p* < 0.001), in agreement with the trend observed in the bead array experiment (data not shown). Dexamethasone treatment significantly reduced the levels of all three mediators in the apical side (*p* < 0.05). However, only CCL5 levels were significantly reduced (*p* < 0.01) in the basolateral side following dexamethasone treatment (Fig. 6D). The JEV-induced disruption to the BBB integrity was significantly reduced by dexamethasone treatment (Fig. 7A). Dexamethasone treatment did not cause a significant change in the levels of infectious JEV particles in both apical and basolateral sides compared to untreated cultures (Fig. 7B).

## 4 Discussion

JE is one of the most important causes of encephalitis globally, with an estimated 67,000 cases annually, for which there is no established treatment (Campbell et al., 2011). An improved understanding of the mechanistic steps during CNS entry of JEV is pivotal to developing targeted anti-inflammatory treatment. This study using an *in vitro* model of the human BBB, has demonstrated that JEV, even at low titres (MOI = 1 in current study *cf.* MOI = 10–20 in published studies), can infect human brain endothelial cells and initiate limited virus production; however, this appears to activate the endothelium, as shown by the release of many host pro-inflammatory mediators from both apical and basolateral sides, leading to increased permeability of the BBB and subsequent viral transmission across it. We found increased levels of pro-inflammatory mediators in the apical side compared to the basolateral side, and increased BBB permeability correlated with levels of several mediators, especially in the apical side. Dexamethasone treatment post JEV inoculation led to a significant reduction in some of the pro-inflammatory mediators that were assessed, and reduced the JEV-induced BBB disruption. The anti-inflammatory action of dexamethasone did not lead to uncontrolled JEV production.

Currently, there is limited understanding of the role of the BBB during JEV infection, including previous conflicting reports from human autopsy studies on whether JEV can indeed infect human brain endothelial cells and astrocytes (German et al., 2006; Iwasaki et al., 1986). Here, we have demonstrated in an *in vitro* model of the BBB that JEV is able to infect human brain endothelial cells and cross the BBB. The results suggest that active production of virions in the endothelium only occurs very early during infection and is not sustained. This initial limited ability to replicate in endothelial cells, followed by an effective shut-down, may explain the differing results from human autopsy studies (*i.e.* inability to access human brain tissue during the early stages of JEV infection). Several studies on *in vitro* rat models have shown that even when high titres of JEV (MOI = 20) are used, there are limited levels of virions in brain endothelial cells compared to BHK21 cells (MOI = 5) (Lai et al., 2012; Chen et al., 2014). We found the limited production of JEV when infected with at an MOI of 1 within HBECs is associated with upregulation of host inflammatory markers and increased permeability of the BBB model, as demonstrated by a significant drop in TEER at 2 dpi. This increased permeability, representing disruption to the barrier integrity of endothelial cells, appears to provide a route for further viral entry into the CNS. As mentioned in the introduction, there are three potential routes for JEV entry into the brain, and we investigated the possibility of the first two routes: passive transport of whole virions across the endothelium, and replication of virus within the endothelium. Our results suggest passive diffusion of the virus across the BBB following barrier disruption is likely, though due to the leakiness of *in vitro* BBB models compared to the *in vivo* BBB, definitive proof of this mechanism in *in vitro* models is difficult to obtain. However, as we have clearly shown infectious JEV particle production in HBECs, and given that barrier integrity is not compromised 2 dpi despite increasing viral titres, it is possible that JEV infects and replicates in HBECs before infiltrating the brain.

It has been suggested that the pro-inflammatory mediators produced in peripheral tissues to control virus replication in animal models of flavivirus encephalitis could be increasing the BBB permeability, thus allowing viral particles and virus-infected leukocytes to enter the brain (Diamond & Klein, 2004). However, it is still not clear which cell types are the key producers of inflammatory mediators *in vivo* that contribute to increased BBB permeability. Here we show using an *in vitro* human co-culture BBB model that cells within the BBB themselves generate this strong, local inflammatory response. Interestingly, a recent study suggests that JEV interferes with the apoptotic pathways in transfected human brain microvascular endothelial cells, perhaps to prolong the period during which viral replication can occur (Al-Obaidi et al., 2017). A previous clinical study and work from a macaque model from our group (Winter et al., 2004; Myint et al., 2014), and several studies on mouse models (Kim et al., 2016; Shukla et al., 2016; Biswas et al., 2010; Chen et al., 2012; Gupta et al., 2010) have shown the importance of pro-inflammatory mediators during JEV infection. In WNV infected mice, detection of the virus triggered the host tissue to induce an innate immune response at the BBB, leading to increased BBB permeability and virus transmigration. However, the detection of the virus in the CNS coincided with a decrease in BBB permeability (Daniels et al., 2014). This biphasic nature of BBB permeability after viral infection suggests that viral and host factors contribute to a potentially differential regulation of the BBB over the course of infection. Furthermore, studies on an *in vitro* rat model have also shown that endothelial barrier can be disrupted by pro-inflammatory cytokines released from JEV infected pericytes and astrocytes (Chen et al., 2014; Chang et al., 2015). Our study reports a detailed account of the levels of soluble mediators released from both the apical and basolateral sides of the BBB, representing mediators released from the apical membrane of HBECs and the sum of mediators released from the basolateral membrane of the HBECs and astrocytes in response to JEV infection. However, passive diffusion, and therefore, some exchange of these mediators between the compartments is likely due to the low TEER of the human BBB model compared to animal models with high TEER (Deli et al., 2005; Patabendige, 2012), and further disruption to the BBB post-JEV infection. The evidence also supports a dual role for brain endothelial cells; maintaining the integrity of the BBB, while contributing to its own disruption by releasing inflammatory mediators during infection. The release of pro-inflammatory mediators by brain endothelial cells is important for attracting and trafficking leukocytes across the BBB during infection. However, the release of these could exacerbate BBB disruption, and open up a pathway for JEV to penetrate the brain.

Although corticosteroids are sometimes used across Asia for patients with JE, their role is unclear. Furthermore, concerns regarding increased viral replication and spread due to the immunosuppressive actions of corticosteroids have limited their use in treating viral encephalitis. One small randomised controlled trial showed that use of corticosteroids was associated with a reduction in the cerebrospinal fluid opening pressure, and number of leukocytes in the spinal fluid, but failed to show any difference in clinical outcomes, partly because of the small number of patients (Hoke et al., 1992; Johnson et al., 1986). The most recent report on a large non-randomised study involving 1199 JE patients, out of whom 737 were treated with dexamethasone did not demonstrate a significant difference in mortality (Sarkari et al., 2012); however, a significant improvement in mortality was observed when the patients presenting with pulmonary oedema were excluded. There is some evidence from animal studies that suggests using corticosteroids as a treatment post infection with viruses such as herpes simplex virus (HSV) or enterovirus 71 (EV71), leads to accelerated virus production and increased mortality. However, the timing of the treatment appears critical (Shen et al., 2014; Sergerie et al., 2007). Early treatment with steroids maybe harmful *in vivo* due to the possibility of corticosteroids affecting the ability of immune cells to clear virus. In clinical observations and animal research on herpes simplex encephalitis (HSE), steroids had no influence on viral replication and dissemination; and retrospective study suggested that they might be beneficial (Sergerie et al., 2007; Thompson et al., 2000; Meyding-Lamade et al., 2003; Kamei et al., 2005; Lizarraga et al., 2013). A multicentre randomised controlled clinical trial on the use dexamethasone in HSE is underway (www.dexenceph.org.uk). Given our observations on the beneficial effects on BBB integrity and no significant increase in JEV production, the potential of dexamethasone and/or other corticosteroids as a treatment against JE may merit further investigation.

Previous work from our group has shown that increased levels of CSF IL6 and plasma CCL5 in JE patients were associated with a poor outcome (Winter et al., 2004). The importance of CCL5 in increasing the adhesion and migration of leukocytes across the BBB has been demonstrated in a rat brain endothelial cell model infected with JEV (Lai et al., 2012); however, in that study there were no permeability changes in the BBB, which was attributed to it being a mono-culture model using a high MOI of JEV for inoculation (MOI = 20). Recently, the same group has shown that rat brain endothelial cells co-cultured with pericytes released IL6 and caused disruption to the BBB, again using a high titre (MOI = 20) of JEV to infect pericytes (Chen et al., 2014). They have also shown the release of IL6, VEGF and MMP2/9 from rat astrocytes infected with JEV in a BBB co-culture model using a high titre of JEV (MOI = 20), which led to a disruption of tight junctions of the BBB (Chang et al., 2015). These studies demonstrated a reduction in the release of cytokines/chemokines when JEV is heat/UV inactivated, suggesting that active virus is required to induce the release of these pro-inflammatory mediators during JEV infection. Intriguingly, a recent study using JEV on transfected human brain microvascular endothelial cells has reported that the integrity of the cell monolayer was MOI-dependent (0.5 to 10). However, JEV replication levels were not quanitified in these cells (Al-Obaidi et al., 2017). Furthermore, another study on mice infected with JEV has also demonstrated high levels of inflammatory mediators (including IL6, CCL5 and CXCL10) in the brain before JEV was detected. Applying brain extracts from these mice on the mouse brain endothelial cell line, bEnd3 significantly increased bEnd3 endothelial cell permeability, but not when the brain extracts were UV-inactivated, suggesting that inflammatory mediators rather than JEV itself were most likely to be responsible for the increased permeability of the BBB (Li et al., 2015). The importance of IL6 during JEV infection and its effect on increasing BBB permeability is in agreement with our previous (Winter et al., 2004) and current work (this study). We used a low titre of JEV (MOI = 1 *cf.* other published studies) in all our experiments, which is much more likely to be close to the lower viraemias thought to occur in human disease, and this was sufficient to induce an increase in BBB permeability through the release of cytokines and chemokines.

## 5 Conclusions

Taken together, our study suggests that infection of HBECs by JEV leads to an initial production of viral particles that activates the brain endothelium. Because we have seen limited JEV production in brain endothelial cells, we postulate that the pro-inflammatory cytokine milieu is the main driving force behind BBB disruption. Results from many *in vitro* and *in vivo* studies confirm that administration of cytokines and/or chemokine either directly on the BBB (de Vries et al., 1996; Wong et al., 2004; Rochfort & Cummins, 2015) or systemically (Saija et al., 1995) leads to an increase in BBB permeability/reduction in TEER, and inhibition/neutralising of these modulators reverses the effects (Deli et al., 2005; Abbott, 2000). Therefore, our data support the hypothesis that release of inflammatory cytokines/chemokines from HBECs and astrocytes in response to JEV infection adversely affects BBB integrity. Indeed, we have shown that dexamethasone treatment following JEV infection reduces the inflammation and restores BBB integrity. The use of dexamethasone and other corticosteroids in clinical practice is limited by fears over their widespread immunosuppressive effects, which might allow uncontrolled viral replication. However, our study has demonstrated that dexamethasone treatment does not lead to increased levels of infectious JEV particles in culture. Targeted immunomodulatory therapies may prove useful in the future.

Our study is not without limitations. Cell culture models of the BBB cannot mimic all the features of the *in vivo* BBB or indeed the neurovascular unit, and there are limitations associated with simulating natural viral infection. Currently no “gold standard” BBB model exists, and relatively low tightness of HBEC monolayers compared to porcine (Patabendige et al., 2013b) or bovine (Dehouck et al., 1990) brain endothelial cells is still a challenge for some studies. As we still lack a complete understanding of the *in vivo* BBB, firmly establishing features of a “gold standard” model is difficult (Helms et al., 2016). Nevertheless, human cell culture models can be powerful tools to investigate host-pathogen interactions in more detail, and can be used to obtain relevant human data when carefully designed to be “fit for the purpose” of predicting human response (Patabendige, 2012). Understanding the complexities of the interactions of neuroinvasive viruses with the human BBB is imperative for developing new therapies. Using a model derived from human brain cells, we have tried to mimic the human condition as closely as possible in laboratory conditions. However, since we have not introduced immune cells into the system, the immune cell-mediated suppression of virus production, and the immunosuppressive actions of dexamethasone on immune cell-mediated response require further investigation. With advanced physiologically relevant multi-cellular flow-based 3D BBB models and increased availability of human material from brain banks and commercial suppliers, future research will be able to build up on these observations and reduce the reliance on the use of animals.

## Appendix A Supplementary data

Supplementary material Image 1
