# Supplementary material for: Brain microvascular endothelial-astrocyte cell responses following Japanese encephalitis virus infection in an in vitro human blood-brain barrier model
Source: Mol Cell Neurosci. 2018 Jun;89:60–70. doi: 10.1016/j.mcn.2018.04.002 (PMC5984247; doi:10.1016/j.mcn.2018.04.002)
Supplement: Supplementary file 1 — Supplementary material [file mmc1.pdf]

Supplementary material

Brain microvascular endothelial-astrocyte cell responses following Japanese encephalitis virus infection in an *in vitro* human blood-brain barrier model

Patabendige et al. 2018

Methods

Immunofluorescence experiments

HBECs/astrocytes were grown on extracellular matrix-coated culture slides and were infected with JEV as described above and immunofluorescence experiments were performed to detect JEV infection. In brief, cells were washed with PBS, fixed with 4% paraformaldehyde for 45 minutes and then permeabilised in 0.1% Triton X- 100 and washed again with PBS. To block nonspecific binding, cells were treated with normal goat serum (NGS) and incubated overnight at 4 °C with primary antibodies: mouse anti-JEV E glycoprotein at 1:20 (Abcam) and rabbit anti-ZO-1, 1:100 (Life Technologies) diluted in PBS containing NGS. Human astrocytes were labelled separately with mouse anti-JEV E glycoprotein at 1:20 (Abcam) and astrocyte marker glial fibrillary acidic protein (GFAP, from Sigma) at 1:400 diluted in PBS containing NGS. Negative controls were incubated with PBS containing NGS. Cells were subsequently rinsed with PBS and incubated for 2 hours at room temperature with secondary Alexa Fluor 488-labelled goat anti-mouse antibody and Alexa Fluor 594-labelled goat anti-rabbit antibody (1:1000 in PBS) for HBEC-5i and Alexa Fluor 488-labelled goat anti-mouse antibody for astrocytes. Cells were washed again for 60 minutes with PBS before

mounting using Fluoroshield with DAPI. Samples were visualised by fluorescence microscopy (Nikon Eclipse 80i) and images were captured by Nikon Digital sight DS-U1 camera using Nikon NIS-Elements BR software.

JEV mRNA extraction and real-time RT-PCR assay

Viral mRNA was extracted from supernatants collected from JEV-infected cells using QIAamp Viral RNA Mini Kit using spin columns (Qiagen) according to the manufacturer's instructions. Real-time RT-PCR assays were performed using the BioRad CFX real-time PCR system with a 96-well configuration using the iScript One-Step RT-PCR kit with SYBR Green (BioRad, CA). The PCR reaction mixture contained 2.5 µl of RNA, 12.5 µl of 2 x SYBR Green RT-PCR reaction mix, 0.5 µl of iScript reverse transcriptase with 20 µM primers in a total volume of 25 µl. Primers for JEV were JE-3F (nucleotide 10726-10750; GGTGTAAGGACTAGAGGTTAGAGG) and JE-3R (nucleotide 10848-10871; ATTCCCAGGTGTCAATATGCTGTT). The RNA was reverse transcribed for 30 minutes at 50 ºC and inactivated at 95 °C for 5 minutes. The products were amplified with 45 PCR cycles, consisting of a denaturation step at 95 °C for 10 seconds and an annealing-extension step at 55 °C for 30 seconds. Melting curve analyses were also performed. Each RNA sample (n = 3 independent samples in triplicates) was also run in technical triplicates. Each run also contained a 7-point standard curve and a no-template control. RNA copy numbers were interpolated from the standard curve and analysed using GraphPad Prism (version 7).

Results

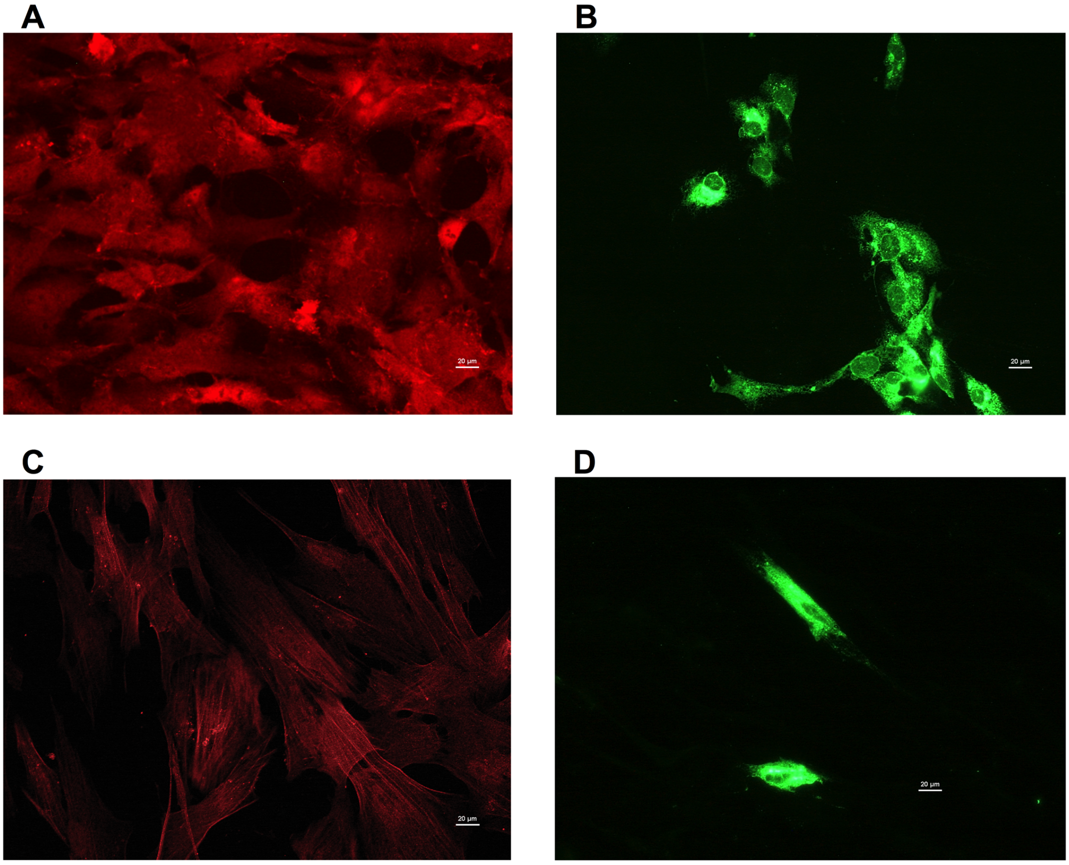

Supplementary Figure 1. Immunofluorescence experiments on HBECs and human astrocytes. The cells were innoculated with JEV and fixed at 3 days post infection (dpi). (A) Control HBEC cultures displaying tight junction protein marker, ZO-1, and (B) JEV infected HBEC with JEV antigen (green) 3dpi. (C) Human astrocytes displaying astrocytic marker, (glial fibrillary acidic protein, GFAP) under control conditions, and (D) JEV infected astrocytes with JEV antigen (green) 3dpi (scale bar = 20µm).

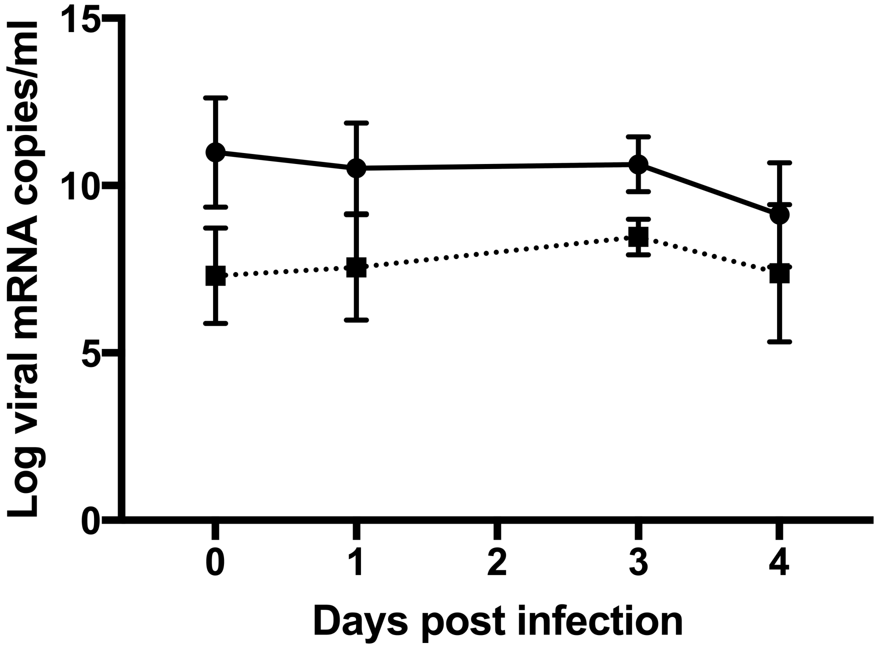

Supplementary Figure 2. Viral mRNA distribution of the human BBB model following JEV infection. Human BBB model was infected with JEV (MOI = 1) from the apical side and viral mRNA was extracted from both apical (continuous line) and basolateral (dotted line) sides at serial time points for analysis by RT-PCR. Significantly higher amounts of mRNA were detected in the apical side compared to basolateral side. Results are mean ± SD (n = 3). Two-way ANOVA, P<0.01 comparing apical and basolateral sides.
